# Has the two decades of research on the gut microbiome resulted in making healthier choices?

**DOI:** 10.1017/gmb.2024.13

**Published:** 2024-12-02

**Authors:** M. Andrea Azcarate-Peril

**Affiliations:** Center for Gastrointestinal Biology and Disease (CGIBD), Department of Medicine, Division of Gastroenterology and Hepatology, School of Medicine, UNC Microbiome Core, University of North Carolina, Chapel Hill, NC, USA

**Keywords:** gut microbiome, diet, vaginal versus caesarean birth, antibiotics, health choices, ultra-processed foods

## Abstract

The gut microbiome is widely recognized for its significant contribution to maintaining human health across all life stages, from infancy to adulthood and beyond. This perspective article focuses on the impacts of well-supported microbiome research on global caesarean delivery rates, breastfeeding practices, and antimicrobial use. The article also explores the impact of dietary choices, particularly those involving ultra-processed foods, on the gut microbiota and their potential contribution to conditions like obesity, metabolic syndrome, and inflammatory diseases. This perspective aims to emphasize the need for updated guidelines and policy interventions to address the increasing global trends of caesarean deliveries, reduced breastfeeding, overuse of antibiotics, and consumption of highly processed foods to counter their adverse effects on gut health.

## Introduction

**Figure 1.**
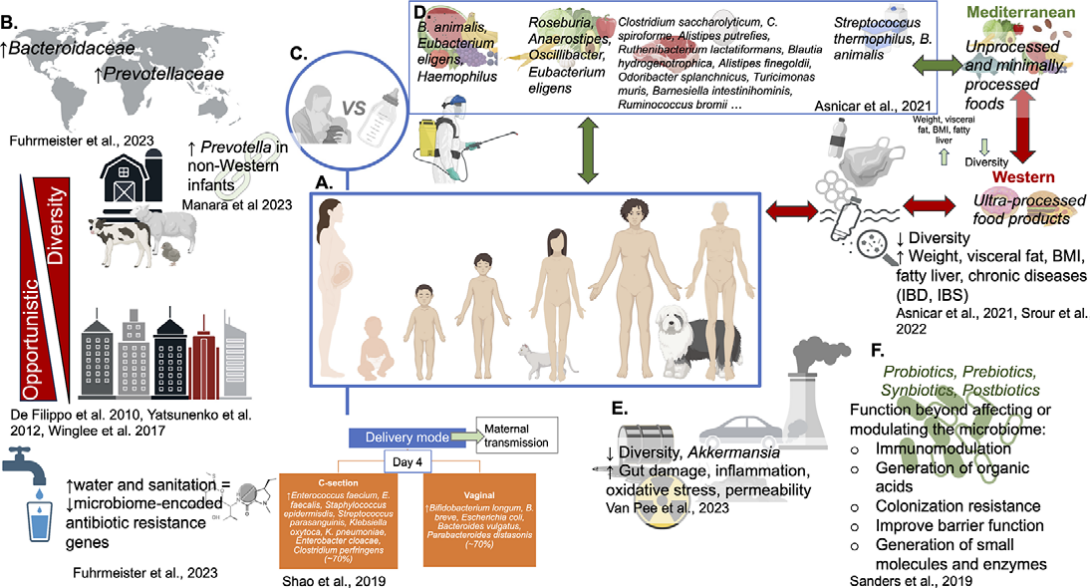
The factors that influence the gut microbiome through life. (A) Life stages and household composition, (B) Geographical region, rural versus urban environments, (C) Delivery mode, breastfeeding, (D) Diet and nutrition, (E) Pollutants, and (F) Probiotics, prebiotics, synbiotics, postbiotics. Based on references: (Asnicar et al., 2021; De Filippo et al., 2010; Fuhrmeister et al., 2023; Manara et al., 2023; Shao et al., 2019; Srour et al., 2022; Van Pee et al., 2023; Winglee et al., 2017; Yatsunenko et al., 2012).

Approximately two decades ago, environmental microbiology methods were adapted to enable the identification of the various components in complex communities of microorganisms, thereby revolutionizing the study of these communities, from terminal restriction fragment length polymorphism analysis and similar methods (Arnold et al., 2016; Liu et al., 1997; Muyzer & Smalla, 1998) to high-throughput sequencing, which in the beginning was dominated by the relatively long reads generated by 454 sequencing (Margulies et al., 2005). Today, the vast amount of data generated by short and long-read sequencing, along with the bioinformatics tools developed to analyze this data, allow us to identify meaningful microbiome responses to nutritional, pharmaceutical, and disease factors in terms of composition and function (Cani & Delzenne, 2011; Ursell et al., 2012). Furthermore, advancements in methodologies for microbiota analysis and their correlation with overall health have significantly improved, allowing us to address technical issues, including, for example, low biomass contamination issues (Eisenhofer et al., 2019; Kennedy et al., 2023), and facilitated more intricate bioinformatic analysis. As I stepped up to the role of editor-in-chief of Gut Microbiome Journal and became aware of the massive amount of research generated by the newest ‘omics technologies, one question resonated: *Have gut microbiome studies, approaches, and capabilities beneficially impacted human lives and the health of our planet?* Several excellent reviews and meta-analysis publications have summarized compositional and functional gut microbiome studies in correlation with its impacting elements (Figure 1). This article aims to present a summary, an updated perspective, or agreement on whether and how, at various stages of life, factors such as mode of delivery, infant feeding, antibiotics, and diet, which are known to have a clear impact on the gut microbiome, have prompted changes in behaviours and guidelines.

## What we know about the gut microbiome in infancy

Although a neonate is first exposed to a rich and diverse microbial community at delivery, there is also indirect exposure to a complex microbial community before birth (Enav et al., 2022). The infant gut microbiome goes through a complex assembly process. It is safe to state that the optimal assembly of the gut microbiome will have a dramatic lifelong impact, with elements like delivery mode, vertical transmission from caregiver to baby, feeding (breast versus bottle), and other environmental sources like family members and pets (Enav et al., 2022; Stewart et al., 2018). From an early study that showed that, even at seven years of age, significantly higher numbers of clostridia were found in children delivered vaginally compared with caesarean-born children, a multitude of studies have confirmed the disruptive effects of C-section on the assembly and evolution of the infant gut microbiota (Sandall et al., 2018). This led to a method of restoring the infant gut microbiota by colonizing newborns with vaginal fluids from the mother, showing at least a partial restoration (Dominguez-Bello et al., 2016; Hourigan & Dominguez-Bello, 2023). Likewise, the evidence on the benefits of breastfeeding is solid, known since the 1970s from a study in Germany that reported that formula-fed babies had decreased abundance of *Bifidobacterium* and increased *E. coli* and neomycin-resistant bacteria (Grutte & Muller-Beuthow, 1979). A longer duration of exclusive breastfeeding has been associated with reduced diarrhoea-related gut microbiota dysbiosis, with effects persisting after six months of age (Ho et al., 2018). The evolution of the infant gut microbiome will continue to be beneficially influenced by a healthy and nutritious diet, access to water and sanitation, exposure to healthy commensal environmental bacteria often found in soil (Seedorf et al., 2014; Sprockett et al., 2018), and minimal exposure to antibiotics, contamination, and pollution.

Over a decade ago, in his best-selling book “Missing Microbes, (Blaser, 2014)”Martin Blaser presented a compelling argument linking the overuse of antibiotics to the depletion of beneficial human-associated bacteria. Early-life exposure to antibiotics has been associated with long-term adverse health outcomes, including childhood asthma, obesity, inflammatory bowel disease, and impaired growth (refer to Table 1 in Tamburini et al. (2016)). Recent research has raised questions about the relationship between autism, hyperactivity disorders, and asthma in familial analysis, suggesting that household co-exposures could potentially confound previous analyses (recently reviewed by Thanert et al. (2022)). Nevertheless, studies using genetically identical and environmentally controlled animal models provide mechanistic evidence supporting a direct link between early-life antibiotics and adverse health outcomes. Hence, while the mechanisms underlying the impacts of antibiotics on chronic health conditions may remain elusive, their effects on the microbiome and the host immune system are likely contributing factors.

## What has changed?

The perceived safety and short-term benefits of caesarean delivery often lead to it being performed without sufficient deliberation. However, potential long-term risks are seldom discussed, and some women opt for this birth method due to a lack of awareness (Antoine & Young, 2020). Moreover, despite extensive evidence of the influence of the mode of delivery on the gut microbiome and overall health (Mitchell et al., 2020; Rios-Covian et al., 2021; Sandall et al., 2018), according to the CDC, the percentage of deliveries by caesarean section increased in the US in 2021 to 32.1% after a decreasing trend from 2012 to 2020 (Osterman et al., 2023). According to the European Perinatal Health Report (Peristat, 2019), the mode of delivery differed markedly throughout Europe from 2015 to 2019, with lower levels of caesarean births (16% to 17%) in most Nordic countries and the Netherlands and higher caesarean rates in Cyprus, Romania, Bulgaria, Poland, and Hungary (>40%). Likewise, although the World Health Organization (WHO) and the United Nations Children’s Fund (UNICEF) recommend exclusively breastfeeding for the first six months of the infant’s life (World Health Organization, n.d.), only 48% of infants 0–5 months of age worldwide are exclusively breastfed. South Asia has the highest prevalence of exclusive breastfeeding (>60%). In contrast, only 26% of infants 0–5 months in North America are exclusively breastfed (United Nations Children’s Fund, n.d.). Notably, most infants born in the US in 2019 started receiving breast milk (83.2%) and continued at one month (78.6%). At six months, 55.8% of infants still received some breast milk, and 24.9% were exclusively breastfed (CDC, 2022).

In 2015, the WHO launched the Global Antimicrobial Resistance and Use Surveillance System (GLASS) as the first step to address antimicrobial resistance (AMR), which globally threatens human health. As of the end of 2022, 127 countries, territories, and areas participate in GLASS. Based on data downloaded from the GLASS Resource Centre (WHO, 2023), the average annual antimicrobial consumption data (Defined Daily Dose [DDD] per 1000 inhabitants per day) from 2015 to 2020 increased on average in the Western Pacific (from 18.3 to 22) and Eastern Mediterranean Regions (from 38.1 to 38.8) but decreased in the European (from 19.1 to 16.2), African (from 35.2 to 23.9), Southeast Asia regions (from 41.4 to 9.5), and the Region of the Americas (from 19.8 to 17.6). We must be cautious with this data as not all regions or countries have yearly data.

## The adult gut microbiome: Are we making healthy lifestyle choices?

A “healthy” (devoid of overt disease (Aagaard et al., 2013)) microbiome is a somewhat vague concept where core functions not carried out by the host are provided by a microbial community capable of maintaining or rapidly returning to its functional composition after a shift induced by diet, disease, or physiological events (Lloyd-Price et al., 2016). After the stable phase is reached in infants (around 31 months), changes to the gut microbiome are rapid and temporary, showing a significant degree of interpersonal diversity, even without disease (Qin et al., 2010). Extensive research has identified major factors that affect the composition of the gut microbiome, such as age, diet, geography, disease, drugs, and exercise, despite interindividual variability. Other factors include smoking, alcohol consumption, gender, and circadian rhythm (summarized in references (Fan & Pedersen, 2021; Gomaa, 2020; Ramos et al., 2022)).

Despite various gut microbiome configurations being linked to well-being, dietary choices significantly influence overall health by impacting the composition and function of the gut microbiome. The recent review by Ross et al. (2024) examined the mechanisms by which Mediterranean, high-fiber, plant-based, high-protein, ketogenic, and Western diets influence the gut microbiome. In agreement with multiple prior studies, the authors concluded that the Western diet is linked to chronic inflammation, obesity, and other non-communicable diseases. Early studies reported correlations between obesity and metabolic syndrome and the gut microbiome. Although most studies agreed that obese individuals have an overall lower microbiota diversity, the results on specific biomarker taxa were inconsistent (summarized recently by Schupack et al. (2022)) and, therefore, unable to predict causation. Later studies on diet and the microbiome have paired increased cohort sizes with more sophisticated and highly sensitive analyses to pinpoint how individual nutrients and dietary configurations impact the microbiome overall and specific taxa, both compositionally and functionally. The PREDICT1 study reported in 2021 (Asnicar et al., 2021) the habitual diet data, demographic information, cardiometabolic blood biomarkers, and postprandial responses to standardized test meals of 1,098 deeply phenotyped individuals. The study examined different food components to create dietary indices, such as the Healthy Food Diversity (HFD) index, which considers food quality and variety. Other indices included the Healthy (hPDI) and Unhealthy Plant-based Dietary Indices (uPDI), which consider the quality and quantity of plant-based foods. The study also assessed the Healthy Eating Index (HEI) to evaluate adherence to dietary guidelines and the alternate Mediterranean diet (aMED) score. Researchers pointed out significant links between the composition of the gut microbiome and these dietary indices, demonstrating the important relationship between diet and overall health.

## Are adults leaning towards health-conscious nutritional choices?

The widely used NOVA system (Monteiro et al., 2019; Moubarac et al., 2014) distinguishes four types of foods: *unprocessed and minimally processed foods* (whole foods modified without adding new substances to extend shelf-life, safety, or palatability), *processed culinary ingredients* (natural ingredients for use in food preparation), *processed foods* (the combination of culinary ingredients added to unprocessed or minimally processed foods), and *ultra-processed foods* (UPF, ready-to-consume, and ready-to-heat formulations, made by combining substances derived from foods with additives, typically through a series of industrial processes). The available information and evidence on the impact of UPFs on gut health is limited due to the numerous confounding factors, including environmental and compositional influences. These factors encompass the impact of specific additives and stabilizers versus the overall impact of the food product itself. Moreover, as stated by the comprehensive review recently published by Whelan et al. (2024), the classification systems and categories for UPFs have been the subject of debate and disagreement. The number of observational studies that have characterized compositional changes in the gut microbiome in response to high consumption of UPFs is limited. One study conducted in Spain reported that alpha diversity decreased in men who consumed higher UPFs and that, also in men, *Bacteroidota* phylum and *Bacteroidia* class had a positive correlation with industrially processed meat consumption (Cuevas-Sierra et al., 2021). However, numerous significant studies have reported the impact on the gut microbiome of specific macronutrients (fat, sugar), additives (dyes, preservatives), emulsifiers, and artificial sweeteners (Srour et al., 2022; Whelan et al., 2024).

Although it may be challenging to distinguish, the evidence of the detrimental effects of UPFs on gut health, either due to their processing methods or the inclusion of harmful additives such as dyes, sweeteners, or emulsifiers known to have detrimental effects on the gut microbiome and overall health, cannot be disputed. A systematic review from 2021 focused on 100 unique studies that estimated UPF levels of consumption in 21 countries (Marino et al., 2021) and reported that the US and the UK had the highest UPF consumption (over 50%), with Italy reporting approximately 10% consumption. Unfortunately, the consumption of UPF has increased significantly worldwide. In the US adult population, the consumption of UPF has increased dramatically from 2001–2002 to 2017–2018 (from 53.5 to 57.0%kcal), while minimally processed foods decreased significantly (from 32.7 to 27.4%kcal) (Juul et al., 2022).

## Conclusion: Time waits for no one

A perspective article that aims to analyze the impacts of well-supported microbiome research on changes in guidelines and regulations may overlook important literature and reach broad conclusions. However, despite this and some seemingly contradictory studies (Is the human gut microbiome sterile at birth? (Kennedy et al., 2023) Are there marine bacteria in the gut? (Offord, 2023)), the central role of gut microbiota on human health is undeniable. Accordingly, the environment, which includes everything that comes into contact with humans and surrounds them, including diet, is arguably the most influential factor in shaping our gut microbiome. Inter-country variation in taxonomic composition significantly exceeds inter-personal variation (Li et al., 2014). Moreover, rural or traditional versus urban environments impact the gut microbiome. These categories are affected by seasons, and the availability of fresh produce in rural communities versus elaborated and seemingly varied foods in urban environments (Davenport et al., 2014; Smits et al., 2017). The continued exposure to environmental cues, including toxic compounds like dietary preservatives and dyes, pollutants, pesticides, heavy metals, and microplastics, as we age can lead to the exacerbation of the characteristics of unhealthy ageing processes, which include the accelerated increase of pathobionts, depletion of beneficial bacteria, and increased inflammation and frailty (De Filippis et al., 2024; Ghosh et al., 2022).

Dietary choices exert one of the most significant impacts on the composition and functionality of the gut microbiome, thereby playing a crucial role in systemic health. Hence, a healthy diet has the potential to extend our quality of life as we age. One avenue to restore the gut microbiome is through personalized nutritional interventions (Duan et al., 2024; Kolodziejczyk et al., 2019) and well-researched probiotics, prebiotics, or synbiotics (Arnold et al., 2018, 2021a, 2021b; Azcarate-Peril et al., 2021; Chey et al., 2020; Hu et al., 2024; Merenstein et al., 2024; Sanborn et al., 2022). However, a comprehensive review of guidelines on UPFs, including emulsifiers, colorants, and other additives, resulting in immediate, impactful regulation, could mitigate their negative impact on the human microbiome and overall health (Brichacek et al., 2024; Whelan et al., 2024), including behaviour (Prescott et al., 2024). Unfortunately, regulators tend to use individual tools to address specific risks rather than coordinated strategies to tackle cumulative harm (Northcott et al., 2023).

Moreover, the existing regulatory policies on diet and nutrition are heavily influenced by the food industry, with few policies directly targeting UPFs. One study reported that from 1983 to 2022, only 25 policy actions were proposed or passed, with eight being federal and 17 being state actions. Of those 25, 22 were proposed or passed between 2011 and 2022 (Pomeranz et al., 2023). Finally, it is striking that UPFs are comparatively less expensive per calorie than unprocessed foods, with respective costs of 0.55 versus 1.45 in $/100 kcal. This cost disparity exists despite the former offering a lower nutrient density (NRF9.3 per 100 kcal: 21.2 versus 108.5) and higher calorie content (higher energy density, 2.2 versus 1.10 in kcal/g).

An emphatic and adequate conclusion for this perspective is the call to action from the Global Research Food Program at the University of North Carolina Chapel Hill: “UPFs are a substantial factor affecting worldwide increases in the prevalence and incidence of obesity and other diet-related, non-communicable diseases. UPFs’ poor nutritional profiles, hyper-palatability (and, arguably, addictive nature), and content of biologically harmful compounds all wreak havoc on health. Policy interventions are needed to curb rising UPF consumption and combat associated negative health outcomes and premature mortality” (Global Food Research Program, 2021).
